# Deficits in Response Inhibition in Patients with Attention-Deficit/Hyperactivity Disorder: The Impaired Self-Protection System Hypothesis

**DOI:** 10.3389/fpsyt.2017.00299

**Published:** 2018-01-22

**Authors:** Thales Vianna Coutinho, Samara Passos Santos Reis, Antonio Geraldo da Silva, Debora Marques Miranda, Leandro Fernandes Malloy-Diniz

**Affiliations:** ^1^Laboratório de Investigações em Neurociência CLínica, Department of Mental Health, Universidade Federal de Minas Gerais, Belo Horizonte, Brazil; ^2^iLumina Neurociências, Belo Horizonte, Brazil; ^3^Quantitative Methods and Predictive Psychometrics Laboratory, Department of Psychology, Universidade Federal da Bahia, Salvador, Brazil; ^4^Brazilian Association of Psychiatry, Rio de Janeiro, Brazil; ^5^Department of Pediatrics, Universidade Federal de Minas Gerais, Belo Horizonte, Brazil

**Keywords:** attention-deficit disorder/hyperactivity disorder, anger recognition, theory of mind, visual attention, facial mimicry, alexithymia, error awareness, inhibitory control

## Abstract

Problems in inhibitory control are regarded in Psychology as a key problem associated with attention-deficit/hyperactivity disorder (ADHD). They, however, might not be primary deficits, but instead a consequence of inattention. At least two components have been identified and dissociated in studies in regards to inhibitory control: interference suppression, responsible for controlling interference by resisting irrelevant or misleading information, and response inhibition, referring to withholding a response or overriding an ongoing behavior. Poor error awareness and self-monitoring undermine an individual’s ability to inhibit inadequate responses and change course of action. In non-social contexts, an individual depends on his own cognition to regulate his mistakes. In social contexts, however, there are many social cues that should help that individual to perceive his mistakes and inhibit inadequate responses. The processes involved in perceiving and interpreting those social cues are arguably part of a self-protection system (SPS). Individuals with ADHD not only present impulsive behaviors in social contexts, but also have difficulty perceiving their inadequate responses and overriding ongoing actions toward more appropriate ones. In this paper, we discuss that those difficulties are arguably a consequence of an impaired SPS, due to visual attention deficits and subsequent failure in perceiving and recognizing accurately negative emotions in facial expressions, especially anger. We discuss evidence that children with ADHD exhibit problems in a series of components involved in the activation of that system and advocate that the inability to identify the anger expressed by others, and thus, not experiencing the fear response that should follow, is, ultimately, what prevents them from inhibiting the ongoing inappropriate behavior, since a potential threat is not registered. Getting involved in high-risk situations, such as reckless driving, could also be a consequence of not registering a threat and thus, not experiencing fear.

The attention-deficit/hyperactivity disorder (ADHD) is traditionally characterized by intense and persistent difficulty in regulating attention and/or hyperactivity behaviors and/or impulsivity, thus producing a significant distress in many areas of the affected individual’s life (1). According to the latest edition of the Diagnostic and Statistical Manual of Mental Disorders, Fifth Edition (DSM-V), ADHD admits three different presentations: predominantly inattentive; predominantly hyperactive-impulsive; and combined. This disorder is highly heterogeneous due to different symptomatology, that includes inattention and hyperactivity/impulsivity, and can be classified according to the severity of symptoms and deficits related as “mild,” “moderate,” or “severe” (2).

Recent studies estimate that the prevalence of ADHD ranges from 6 to 7% among children and adolescents, 5% among young adults (3), and 3% among older adults (4). However, despite descriptive statistics found in literature, there is no real evidence of this disorder having increased significantly regarding the number of cases over the past three decades (5).

Since ADHD is considered a lifespan disorder, the impairments vary across age. In childhood, ADHD is related to learning disabilities (6), frequent conflicts in sibling relationships (7), and difficulty interacting with other children (8). In adolescence and/or adulthood, individuals with ADHD have a higher chance of being involved in car accidents and reckless driving (9), especially for those with comorbidities (10); getting in trouble with the law (11, 12); presenting inconsequential sexual behavior (13, 14); as well as frequently changing jobs (15), which leads to the occupation of positions with lower social prestige (16), and lower income (17). Later in life, several burdens, such as impairments on financial and social well-being, may be identified (18). ADHD represents a greater risk for earlier mortality, regardless of age, mainly caused by unnatural events such as accidents (19).

Children with ADHD tend to be considered by their peers as intrusive, irritating, and generally aversive (20), which causes them to have problems maintaining friendships (21). These social deficits remain in adolescence (22). Due to the violation of behavioral norms and expectations, the externalizing behavior mediates the relationship between ADHD symptoms and peer rejection (23). Association between peer rejection and ADHD symptomatology goes both ways, since ADHD symptoms at age of 4 predicts more peer rejection at the age of 6, and also, peer rejection at the age of 4 predicts more hyperactivity symptoms at the age of 6 (24). Social exclusion and self-regulation are reciprocally regulated as well: the limited ability to suppress impulses in favor of reaching goals predicts social exclusion and *vice versa* (25). A common behavior associated with the negative peer evaluation of children with ADHD is excessively blaming peers for their inabilities, when dealing with negative outcomes (26). This condition results from a positive illusory bias, in which individuals with ADHD overestimate their competencies when feeling threatened in a competitive situation (27), which may lead to their own ostracism.

Studies support that inattention and hyperactivity/impulsivity symptoms in ADHD are distinguishable but substantially correlated (28). Recently, a study based on three different and independent data sets, collected among children, adolescents, and adults, established that there is a causal path from inattention to hyperactivity/impulsivity, concluding that clinical interventions focused on the former will probably affect the latter, but not the other way around (29).

Inhibitory control is a key construct for understanding symptoms in ADHD (30). Several studies have pointed to remarkable impairment in inhibitory control in patients with ADHD (31, 32). Inhibitory control has been defined as the ability to deliberately suppress or interrupt the expression of cognitive, emotional, or behavioral responses (33–35). According to Barkley (30), such inhibition is composed of three separated and overlapping processes, responsible for: 1. inhibiting a certain unwanted behavior, creating a delay in the final answer; 2. stopping an answer in progress, being sensitive to error and changing the course of an answer which will prove unsatisfactory; 3. resisting the distraction that can occur during the delayed response, allowing oneself to carry the decision of changing the strategy until the end.

Some authors argue that inhibitory control might have an emotional foundation in which the conflict between two or more stimuli results in an aversive experience that provokes a negative emotion, leading the individual to exert control in order to resolve the conflict. In other words, the conflict-related emotion is a necessary precursor for control (36). In typical individuals, increasing negative emotions enhances cognitive control (37). Here, we will approach inhibitory control in this emotional perspective, applied to social contexts.

Recent studies have established that inhibitory control can be considered as a modular construct (38), with at least two different components that have different electrophysiological correlates (39). “Interference suppression” is the component related to resisting irrelevant or misleading information, whereas “response inhibition” refers to the capacity of withholding a response or overriding an ongoing action (40, 41).

In this paper, we will address problems regarding the “response inhibition” component in individuals with ADHD, especially in regards to the behavioral regulation in social contexts. Our purpose is to discuss how processes like visual attention and recognition of facial expressions are involved in a Self-Protection System (SPS), which enhances error awareness and inhibition of inadequate social behaviors. We argue that an impaired self-protection system (ISPS) is ultimately what causes perseverance of improper behaviors related to impulsivity/hyperactivity in individuals with ADHD.

## Emotion Recognition in ADHD

Human communication is multimodal, occurring through different channels of communication, such as facial and corporal expressions, speech, and prosody (42). The correct emotion recognition through facial expressions is critical to social adaptation because, among other things, it stimulates self-monitoring (43).

Individuals with ADHD have difficulty recognizing emotions in facial expressions (44, 45), an endophenotype shared with autistic patients (46). These difficulties are accentuated when negative emotions (such as anger) are concerned and may partially explain relationships problems with family members and peers (47, 48). Children with ADHD tend to take more time and make more mistakes when trying to recognize emotions such as sadness, disgust, or anger (49).

According to a meta-analysis, deficits in recognition of anger and fear in facial expressions have been observed in children with ADHD (50). Highly hyperactive individuals are usually less likely to recognize fear, while individuals with the predominantly inattentive type of ADHD are less likely to identify anger (51). There seems to be a correlation between attention deficits and difficulty to identify anger in children with ADHD (52), and those problems with the recognition of emotions in facial expressions may actually result from visual attention deficits (53).

A neuroimaging study analyzed hemodynamic responses to expressions of happiness and anger in boys with ADHD, and it concluded that they have a lower hemodynamic response when facing the expression of anger (54). Young adults with ADHD also remained less sensitive to anger expressions (55), had impairments in recognizing anger in prosody (56) and had problems adequately responding to anger (57).

## Fear the Anger: The SPS

Anger is one of the seven universal facial expressions of emotion (58) and, therefore, has very specific markers, mainly characterized by lowered and joined eyebrows, wide eyes, and upper eyelids pressed against the eyebrow —a kind of a “stare” look—along with tight and heavily strained lips (59). According to studies carried out in different cultures around the world, this pattern of facial contraction, despite the cultural influences (60), is quickly and easily identified by all subjects.

Evolutionary social psychology describes the mechanisms to detect any possible threat and properly respond to it (61–65). The “Self-Protection System” (SPS) (66) is one of them, and its function is to identify social cues that may indicate possible risk or intention of damage, responding to this threat perception with the activation of a cognitive and affective response, which facilitates escape (67).

An expression of anger is evaluated by that system as a possible signal of violent intent, and it leads to a fear response (68). At the cognitive level, the potential threat (in this case, anger expression) triggers an immediate response of “stop, look, and listen” (69), disrupting the ongoing action. And it is already known that the main category that elicits anger in a daily basis is, in return, “other people,” which highlights that it is an emotion with an important social trigger (70).

An expression of anger can be a social sign of rejection of the other (71), disapproval, and/or intention of harm (72, 73). One person in a group expressing anger toward another immediately causes the targeted individual of that anger to feel excluded and motivated to act in a way so as to be accepted once again by the group (74). This happens because being rejected usually increases the motivation to reconnect to the group (75, 76).

An angry face can be tracked much faster in a crowd of neutral faces, in comparison to a happy face (77–82). This shows that, under normal circumstances, individuals tend to prioritize anger to the detriment of other emotions, and its identification triggers fear, which is critical to behavioral control (83). The probable cause for this is that anger is an emotion strongly associated with the intention of causing harm to something or someone, and the sooner identified, the better it is concerning survival fitness. This phenomenon is called the “Anger Superiority Effect” and occurs both in children and adults (84).

Children in kindergarten seem to achieve a better performance in Go/No-Go tasks when they experience negative emotions, probably because those emotions lead to a more focused and attentive behavior, oriented toward problem-solving and reducing the chance of committing mistakes (85). Studies using emotional Go/No-Go tasks have concluded that emotional processing interferes with inhibitory control (86, 87).

Physiological studies indicate that the hypothalamic–pituitary–axis (HPA) plays a fundamental role in stress response by promoting behavioral and peripheral changes capable of maximizing the body’s ability to adjust its homeostasis and increasing the chances of survival through the release of glucocorticoids, mainly cortisol (88). It has already been proved that fear is one of the main triggers for cortisol once the response of the HPA axis is more prominent whenever an individual experiences fear in response to a stressor (89).

It was reported that cortisol administration may improve inhibitory control in healthy adults on a Go/No-Go task (90). In another study, results indicated that the administration of a cortisol antagonist eliminated the positive effect of the hormone related to inhibitory control observed in healthy participants (91). A third study carried out with women, with and without borderline personality disorder, showed that a single cortisol administration improved inhibitory control for both groups (92).

Children with ADHD seem to present lower levels of diurnal cortisol, in comparison to those without the condition, and treatment with atomoxetine may help normalize these levels (93). Also, children and adolescents with ADHD, especially those with Defiant Oppositional Disorder or Conduct Disorder as comorbidities, presented low HPA responsiveness, having hyporesponsiveness to stressful situations, which may result in impulsive and/or defiant behaviors (94), as well as deficits in emotional regulation and aggressiveness inhibition (95). Pharmacological treatment with methylphenidate helps normalize the HPA alteration in children with ADHD (96).

## The ISPS Hypothesis

Some authors emphasize the need to consider other processes for a more comprehensive understanding of ADHD, highlighting the importance of those related to emotion and social cognition (97). In an attempt to unify all of these aspects, we developed the “Impaired Self-Protection System” (ISPS) hypothesis, which explains response inhibition deficits in social contexts, more specifically, why individuals with ADHD seem unable to regulate their inadequate behavior even in the presence of disapproving social cues.

Children with ADHD have visual attention deficits (98), which compromises visual processing speed and sustained visual attention (99). We already know that visual attention deficits impair the ability to perceive facial expressions (100, 101). Emotional perception is often considered a low perceptual process necessary to decode affective cues or identify outgoing emotional information in the environment (102). It is also the first step to Theory of Mind (103).

Theory of Mind refers to the natural ability to assertively infer other people’s beliefs and desires, and to use this information to make assumptions and predict their behavior (104, 105). Several studies demonstrated that children with ADHD have a deficit in Theory of Mind (106–108). A recent meta-analysis considered the Theory of Mind impairments observed in patients with ADHD halfway between those observed in individuals with typical development and in individuals on the autism spectrum (50).

As mentioned before, Anger Recognition is the first step to activating the SPS, and Theory of Mind is responsible for properly interpreting that emotion (109). If there is impairment in Theory of Mind, an expression of anger may go unnoticed or be misjudged, and ultimately, the fear response that should have been triggered in that situation will not arise. Consequently, the improper behavior that induced that anger in the first place will persist instead of being discontinued.

A recent study partially corroborates this hypothesis by establishing that patients with ADHD, in addition to their difficulty recognizing anger, show a reduced ability to inhibit responses in the emotional Go/No-Go task, and this difficulty is more pronounced when the stimulus is a face expressing anger (110). This hypothesis aligns itself with the concept of Deficient Emotional Self-Regulation, characterized by deficits in the ability to inhibit inappropriate behavior in the face of certain emotional display (111), which, in the case of patients with ADHD, is anger.

Additionally, there are other processes indirectly involved in the activation of the SPS that seem to be impaired in patients with ADHD. Hereupon, we will present them and, subsequently, make an effort to unravel the possible connections between them and the other variables discussed so far.

## Facial Mimicry

“Mimicry” can be defined as the tendency to imitate facial, vocal, or postural expressions of the person who we are interacting with (112). There are four characteristics that define “emotional mimicry”: 1. both people present the same emotional expression, although not necessarily through the same communication channel; 2. this expression occurs in a short window of time, usually within the first second; 3. the expression of the “mimic” is linked to the expression of the imitated person; 4. the mimetic expression consists of a sharing of the original expression, rather than a reaction to the original expression (113).

Facial mimicry favors the emotional experience in itself, which facilitates the recognition of the emotion of the other (114). It is an important tool for reconnecting with a group after social exclusion (115).

There is evidence that the corrugator supercilli muscle, fundamental to creating facial expressions of negative emotions, such as anger and fear, displays electrical activity after only 100 ms of the perception of a mistake. That reaction is associated to the concept of “error awareness,” defined as the tendency to slow down responses after perceiving a committed mistake or a received punishment, increasing self-monitoring and proceeding more cautiously in order not to commit further mistakes (116).

Some neuroimaging studies have already identified brain circuits involved in facial mimicry (117). Specifically for the anger mimicry, it is known that lesions in the right frontal cortex decrease its proper expression (118). It is also known that the right frontal cortex plays a key role in inhibiting unwanted behavior (119, 120). In children with ADHD, for example, abnormal functioning of the right frontal cortex has been associated with deficit in inhibitory control (121, 122). Among children, adolescents and adults a lower cortical thickness in the right upper frontal gyrus has been correlated to the severity of this disease (123).

Considering that one of the main regions involved in the inhibitory control is also responsible for anger mimicry, we could assume that individuals with ADHD present difficulties in facial mimicry, which would hamper their ability to simulate and infer emotions of others. But, so far, only one study has investigated the relationship between ADHD and facial mimicry (124), and it found no association between both. However, we must consider that the age group of participants were very limited (6–7 years old), and differences might have been undetectable due to their development stage, which highlights the need for further studies.

## Emotional Awareness and Alexithymia

The process of observing, identifying, discriminating, and evaluating one’s emotions is called Emotional Awareness (125). In contrast, Alexithymia refers to the inability to access and nominate those emotions and thus, is associated with a deficit in the self-consciousness of the emotional state (126, 127), as well as in the recognition of other people’s emotions (128, 129).

Alexithymia has also been associated to impairments in the processing of threat-related facial expressions, emotion recognition (130), and to reduced anticipation of negative emotional events (131). The difficulty in labeling the emotions of others in alexithymic individuals could be explained by a reduced neural activity in ventral striatum and in frontal, temporal, and occipital cortex in response to brief negative emotional facial expressions (132). Longer reaction times are presented when labeling angry and fearful faces, indicating that they were slower in labeling negative emotions (133). Interestingly, the practice of mindfulness focused on emotional awareness seems to enhance neural sensitivity to errors (134), which might also have an impact on behavioral regulation, leading the individual to be more cautious and avoid committing further mistakes.

There is evidence that children with ADHD show low levels of emotional self-awareness, and it is associated with externalizing behavior, in this case, opposition and challenging behavior in response to some perceived provocation (125). Alexithymia in children with ADHD has been correlated to hyperactivity and to impairments in inhibitory control (135). In adults with ADHD, it has been closely associated with difficulty in accepting their own emotions (136). A preliminary study held with adults that presented ADHD and alexithymia demonstrated that related symptoms improved significantly after pharmacotherapy with psychostimulants (137).

## Overview

**Figure 1:**
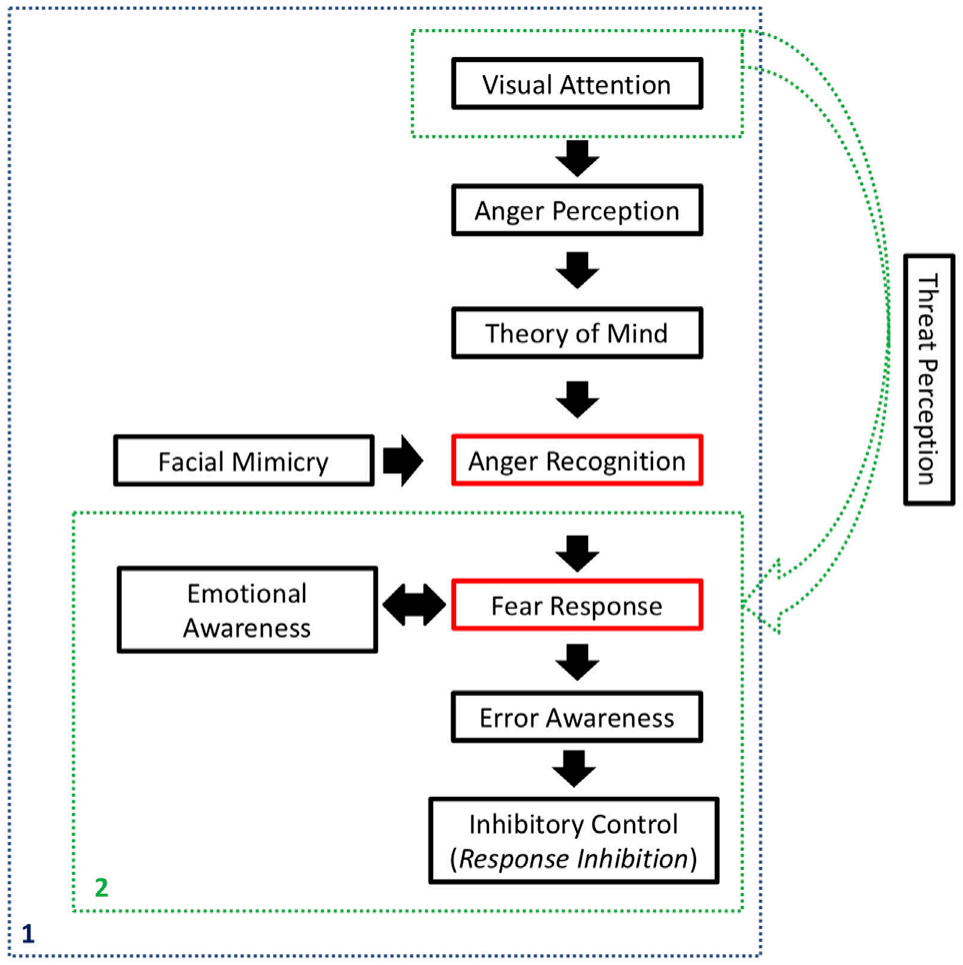
Synthesis of the processes involved on the Self-Protection System (SPS). In blue (1), activation of the SPS in the presence of social inputs, related to anger expression. In green (2), possible activation of the SPS in non-social contexts, when a stimulus is registered as a potential threat. Red rectangles refer to the main components of the SPS, whereas black rectangles refer to processes that affect SPS activation and final response inhibition.

As shown in Figure 1, we began our argument by discussing how impulsive/hyperactive behaviors in ADHD might be a consequence of inattention. Even after committing a mistake, error awareness should help an individual to inhibit an ongoing action and override it. In simple Go/No-Go tasks, there are emotional processes that affect an individual’s performance, but ultimately, he depends on his own cognition to regulate his responses. In social contexts, however, there is a series of social indicators that should help that individual to perceive his mistakes and inhibit inadequate behavior. Even in the presence of those cues, individuals with ADHD seem to persist on improper behavior, which causes them to be rejected by groups.

We continued by explaining how social disapproval of one’s inadequate behavior is usually expressed as an angry expression and how visual attention is important in order to perceive that expression. That perception should be interpreted by the individual using Theory of Mind, in order to recognize that the perceived stimulus is an angry face, and that it is directed to him or her. Facial mimicry will help in the emotion recognition process, by internally simulating the observed expression.

Subsequently, anger recognition should be registered as a potential threat to the individual, allowing the SPS to take action, triggering a fear response. Emotional awareness, underpinned by social cues, will be a key to accurately experience and interpret those emotions, internally and externally. Ultimately, the SPS would lead that individual to inhibit his own behavior, discontinuing the action that had caused social disapproval in the first place. An adequate Theory of Mind and emotional awareness would be critical for interpreting the whole situation and establishing a cause and effect relationship, as well as regulating one’s own emotions and evaluating the appropriate outcome.

All of that represents a whole cognitive circuitry that starts with visual attention and ends with inhibitory control. In individuals with ADHD, however, all of those functions seem to be impaired, impeding the SPS to be set in motion. As a consequence, social behavior is not properly regulated, resulting in social exclusion and worsening the patient’s condition.

We argue that the link between the ISPS and the deficits in inhibitory control, specifically the “response inhibition” component, may be explained by reduced error awareness, since the ability to perceive their own mistakes is fundamental to individuals, in order to control their own behavior (138). Being aware of mistakes committed in a certain context enables the individual to adopt a more cautious behavior in the future, minimizing chances of recurrence (138).

According to a recent meta-analysis, patients with ADHD do not present adequate “post-error slowing,” which is the natural reduction in response times after identifying one’s error. In other words, these patients do not present the tendency to be cautious in order to avoid committing further mistakes (139). Another study indicated that children with ADHD not only committed more errors in Go/No-Go tasks but they were also less aware of the mistakes they committed (140). Some authors suggest that this reduced error awareness plays a key role in the behavioral regulation in individuals with ADHD, precisely because they cannot correctly identify when they are displaying inadequate postures in a particular context (141).

We argue that this reduced error awareness, specifically observed in social contexts in which a child with ADHD does not understand that he or she is behaving inadequately, happens because of problems in the SPS.

It is important to note that an inadequate social response might not only be related to an impulsive/hyperactive behavior but might also be a simply *faux pas* or an improper response due to not understanding a given social rule. Either way, an individual should perceive his mistake and override his action, even trying to compensate for it.

The SPS concept as proposed by evolutionary psychologists specifically designates anger recognition as the stimulus that put that system in motion. However, we theorize that in non-social contexts, other stimuli could be registered as a potential threat, triggering a fear response and leading to response inhibition as well. Other researchers have proposed the risk-as-feelings hypothesis, discussing that emotional reactions to risky situations often account more to decision-making than cognitive assessment of those risks, driving behavioral responses even if it means resisting cognitive interpretation of consequences (142). It is possible that inattention and SPS-related deficits prevent individuals with ADHD from perceiving other sorts of threat, and/or experiencing the fear response that should follow, making those individuals more likely to assume higher-risk conducts. In that perspective, our hypothesis might explain response inhibition deficits in the absence of social inputs, in contexts where ADHD individuals seem to present problems as well, such as reckless driving, inconsequential sexual behavior, and breaking the law.

Interestingly, pharmacological treatment for those patients, particularly the use of methylphenidate, seems to improve Theory of Mind (143, 144) and emotion recognition (145), especially the ability to recognize anger (45). After 12 weeks of treatment with methylphenidate, there seems to be an improvement in the ability to recognize emotions of anger and sadness in children with ADHD (146). This may explain the prompt and effective decrease of dysfunctional behavior in patients with ADHD in response to that drug and corroborate to elucidate the role of emotion recognition in behavior regulation.

## Limitations and Future Research

We must clarify that we do not intend to cover all the complexity of ADHD with the hypothesis here presented. It was outlined based on research and evidence available so far in literature. Therefore, we recognize and emphasize the need for empirical data that might support our hypothesis.

It is important to note that inhibitory control varies significantly depending on the context (147), which implies that this hypothesis does not explain all the possible alterations in inhibitory control in ADHD patients. We narrowed it down to the context of personal interactions, which are permeated by facial expressions of emotions, and we tried to explain the persistence dysfunctional behaviors that occur in those circumstances. In that perspective, a child with ADHD persists in a given behavior that displeases other people because he or she does not recognize anger properly and, therefore, does not realize he or she is being unpleasant or annoying.

Among the several aspects to be further investigated, the “Anger Superiority Effect” has already been analyzed in several psychiatric disorders, such as in Asperger Syndrome (148), generalized anxiety disorder and panic disorder (149) but never in any subtypes of ADHD. Studies in that direction will be important to assessing their ability to perceive anger in a crowd.

In addition to this, the degree of dependence between visual attention and emotional perception is still controversial (101), and so, it is important to develop paradigms capable of assessing that relation in children with ADHD.

As for the deficits in relation to Theory of Mind, most studies do not clarify whether they are related to the affective dimension (theorizing about the affections of others) or to the cognitive dimension (theorizing about thoughts and intentions of others) of that construct, as recently described (150). This type of study would not only refine our hypothesis but also give a foundation to more specific interventions.

There is also a need to clarify whether children with ADHD present a subjective response of fear when facing images of anger expression, which could demonstrate a more specific impairment of the SPS. It would also be important to analyze if there is evidence of alexithymia in patients with ADHD specifically related to fear. Furthermore, it would be interesting to verify if the induction of fear could affect inhibitory control in patients with ADHD. In regards to facial mimicry, studies involving children of other age groups would be important in order to assess if there are any alterations involving anger mimicry or not.

We should investigate if the difficulty in recognizing anger in ADHD patients is restricted to facial expression, since anger can be communicated also through body muscle contraction (151), posture (152), gait (153), and voice (154). Studies focused on this will be necessary to verify if deficits in anger recognition are also present throughout other communication channels, thus suggesting a much more difficulty in emotional recognition.

Clinically, it will be important to investigate whether psychological interventions focused on training recognition and adequate response to anger expressions would significantly improve inhibitory control. It creates a promising field to further investigate ADHD and possibly characterize differences and similarities between gender, age, and subtype of ADHD. Even though our hypothesis has been developed to approach ADHD deficits, it is possible that it might apply to other psychiatric disorders. For instance, response inhibition deficits have been also observed in patients with schizophrenia (155, 156), as have been difficulty in emotion recognition (157, 158). Further research would be necessary in order to analyze if other processes involved in the SPS are also impaired in these patients.

Since we are presenting a novel hypothesis, alternative explanations should be assessed experimentally. Some core deficits in ADHD, as the primary attentional deficit, sensation seeking behavior, and general impulsiveness traits could also explain the lack of ability in perceiving inappropriate social responses. Future studies controlling for inattention and inhibition problems should be carried out using experimental design. The emotional Go/No-Go task (110) may be used with a bigger sample contemplating all three ADHD’s subtypes, and all basic emotions. That might better demonstrate the deficits in inhibitory control specifically when faced with an angry expression.

Our hypothesis might also lead to a better understanding of the inhibitory control mechanism in typical individuals. Individual differences in regards to emotion recognition have been observed in healthy individuals, both in children (159) and adults (160). Those differences are associated with anatomical and physiological differences (161–163). It is possible that different activation of the SPS in typical individuals also explain individual differences in response inhibition, but that it just a possibility not yet supported by empirical evidence, since we are discussing a novel hypothesis.

The purpose of this paper was to present and discuss a plausible theoretical explanation for the problems in inhibitory control, specifically in the “response inhibition” component, observed in patients with ADHD, addressing them as a consequence of an ISPS which does not function properly because of primary visual attention deficits.

We have made an effort to take social, clinical psychology, and evolutionary perspectives into account. Empirical investigation, however, is necessary in order to find evidence that supports the ISPS hypothesis. Ultimately, we hope we have contributed to the efforts of better understanding ADHD and connecting the knowledge gathered so far by the scientific community.

## Author Contributions

TC carried out the literature review and elaborated the initial review that gave rise to the basic structure of the hypothesis. SR contributed to the theoretical basis and discussion of the proposal, besides the semantic and grammatical revision. DM guided the article, contributing to the theoretical basis and discussion of the proposal. AS contributed to the theoretical basis and dicussion of the proposal. LM-D guided the article, contributed to the theoretical basis and discussion of the proposal.

## Conflict of Interest Statement

The authors declare that the research was conducted in the absence of any commercial or financial relationships that could be construed as a potential conflict of interest.
